# 

**Published:** 1906-04-21

**Authors:** 


					the hospital.
^ ArBIL 21st? 1906-
OSELTA Lr
No. 1,021. ^ol, XL [aReSpfper!] Saturday,
April 21st, 1906.
itf/CH HOSRLXJUb
[SabsCsreePpi.06noTerms] PRICE THREEPENCE

				

